# Will Cape Verde attain COVID‐19 herd immunity? Progress, Challenges, and Recommendations

**DOI:** 10.1002/puh2.103

**Published:** 2023-07-12

**Authors:** Deborah Oluwaseun Shomuyiwa, Morolake Ibukunoluwa Otitodun, Tobechukwu Innocentia Anyanwu, Muhsinah Adesewa Abdulwasiu, Alhaji Umar Sow, Maria de Fátima Carvalho‐Alves, Maria da Luz Lima Mendoca, Goodness Ogeyi Odey

**Affiliations:** ^1^ Faculty of Pharmacy University of Lagos Lagos Nigeria; ^2^ Faculty of Pharmaceutical Sciences University of Ilorin Ilorin Nigeria; ^3^ Faculty of Pharmaceutical Sciences University of Port Harcourt Port Harcourt Nigeria; ^4^ Dora Akunyili College of Pharmacy Igbinedion University Okada Nigeria; ^5^ College of Medicine and Allied Health Sciences University of Sierra Leone Freetown Sierra Leone; ^6^ Department of Research Science and Training National Institute of Public Health Praia Cabo Verde; ^7^ Department of Public Health College of Medicine University of Calabar Calabar Nigeria

**Keywords:** Africa, Cape Verde, COVID‐19, health response, healthcare system, vaccination, pandemic

## Abstract

The COVID‐19 pandemic has exposed the vulnerabilities of Cape Verde's healthcare system, prompting a swift and effective response. This article examines Cape Verde's comprehensive approach to the pandemic, focusing on epidemiology, the healthcare system, and the vaccination campaign. Recognizing the critical role of COVID‐19 vaccination in global efforts, Cape Verde has implemented strategic measures to achieve herd immunity. Although the country has achieved relatively high vaccination coverage in Africa, it still falls short of the necessary threshold. Challenges, including vaccine hesitancy, viral mutations, and supply chain insecurities, must be addressed. Sustained efforts, involving community participation and improved vaccine availability, are crucial. Furthermore, a robust pharmacovigilance system is essential for ensuring vaccination safety and enhancing health outcomes.

## INTRODUCTION

Cape Verde, also known as the Republic of Cabo Verde, is a captivating island country located off the coast of Senegal in West Africa [1]. With a population of 556,000 people, Cape Verde relies heavily on tourism and hospitality, making it a vibrant hub of cultural exchange and interaction. Prior to the COVID‐19 pandemic, the tourism sector played a vital role in the country's economy, contributing to 18% of all international tourism receipts in Western Africa and generating 169 million US dollars, which accounted for 8.7% of its GDP in 2020 [2]. However, the outbreak of the COVID‐19 pandemic had a devastating impact on Cape Verde, disrupting livelihoods and causing loss of lives. Despite fully reopening in 2022, the country faced significant challenges, including reduced tourism investments, which had a detrimental effect on the local economy and its reputation as a thriving tourism destination [3]. Moreover, the pandemic had far‐reaching consequences for Cape Verde's healthcare system, compounding the existing challenges faced by the country.

The COVID‐19 pandemic exposed the vulnerabilities of public health systems across Africa, including Cape Verde. In response, travel restrictions, social distancing measures, risk communication campaigns, and the implementation of protective measures such as face masks, respiratory hygiene, and vaccinations were enforced to combat the spread of the virus [4]. Notably, Cape Verde has made remarkable progress in its vaccination campaign, boasting the third‐highest vaccination coverage in Sub‐Saharan Africa, with over 50% of its adult population now fully vaccinated [5]. The article examines Cape Verde's response to the COVID‐19 pandemic focusing on its epidemiology, healthcare system, and vaccination campaign and offers valuable recommendations in achieving herd immunity.

## EPIDEMIOLOGY OF COVID‐19 IN CAPE VERDE

The COVID‐19 case cascade in Cape Verde began with the first confirmed case reported on March 20, 2020, on Boa Vista Island. This was after the World Health Organization (WHO) categorized it as a pandemic disease on March 11, 2020 [6]. As of December 2022, Cape Verde recorded over 63,000 confirmed cases and 400 deaths [7]. Following the announcement of the first imported case, stringent restrictive measures were announced to control the pandemic spread. This included the declaration of a state of emergency, which took effect from March to May 2020 and extended three times [8]. The infection spread steadily, and lockdown measures were put in place. However, after measures were lifted, there were high increases in cases [5]. Strategies such as limiting contact, social isolation, contact tracing, and vaccination were adopted to manage the pandemic. By January 2021, the COVID‐19 attack rate was at 2.6% of the population [9]. In 2022, there was a resurgence of cases attributed to the easing of restrictions, the evolution of the novel virus strains, and the lack of vaccines at the time. The COVID‐19 infection had a high transmission rate, with approximately 8% of the population infected at the end of 2021 [1]. This prompted nationwide response measures, including public health measures and strict lockdown and entry bans.

With the commencement of vaccination in 2021, African countries experienced difficulties with unequal vaccine distribution and allocation, inadequate vaccine storage, vaccine hesitancy, connectivity issues, and challenges in service delivery [10]. Cape Verde surpassed expectations by achieving its vaccination target in 2022, even though the majority of African countries had achieved less than a 5% COVID‐19 vaccination rate [5]. As of March 18, 2022, a total of 724,785 COVID‐19 vaccine doses had been administered in Cape Verde, equivalent to approximately 130.95 doses per 100 people [7]. Vaccination efforts continued with the goal of achieving 70% vaccination coverage by June 2022, in‐line with the target set by the WHO.

## VACCINATION IN CAPE VERDE

In March 2021, Cape Verde received 267,293 doses of AstraZeneca and Pfizer vaccines through the COVAX initiative [11, 12]. Subsequently, the United States partnered with COVAX and donated 300,690 doses of Pfizer vaccines and 100,100 doses of Moderna vaccines in October 2021 [13]. China also contributed 50,000 doses [14]. These donations allowed Cape Verde to swiftly begin vaccinating priority groups [15]. By November 2021, Cape Verde became the fourth African nation to achieve the WHO's global goal of vaccinating 40% of its population by the end of 2021, with approximately 236,000 complete doses administered [16]. This success was attributed to the availability of vaccines, dedicated healthcare providers, committed leadership, and community involvement [16].

However, as of August 2021, only 12% of the population had been fully vaccinated [17]. By September 25, 2022, the number of doses administered had reached over 859,000, with around 300,000 individuals fully vaccinated, representing approximately 60% of the population [7, 18]. To achieve the target of vaccinating 70% of the population and increasing the number of immune individuals, further efforts are needed. This includes encouraging greater community involvement, training additional medical personnel, and implementing effective policies.

## ACHIEVING HERD IMMUNITY

Herd immunity offers indirect population protection from infectious diseases through vaccination or immunity developed through previous exposure to infection [19]. Herd immunity offers a very low probability of transmission of the virus. However, the percentage of the population that needs to be immune to reach the herd immunity threshold varies depending on the specific diseases. Herd immunity, when achieved, will offer indirect protection against COVID‐19, just like every other infectious disease, to susceptible community members [20, 21].

Cape Verde, the smallest country in West Africa with a population of approximately 500,000, has been working toward achieving herd immunity through vaccination. Herd immunity is achieved when one infected person in a population causes less than one secondary case on average, corresponding to the adequate reproduction number (*R*_e_) [19]. The basic reproduction number (*R*_o_) for COVID‐19 ranges from 2 to 6, with various values in different nations, including 2.57 in Cape Verde [22]. Because the herd immunity threshold is defined as 1 − (1/*R*_o_), the appropriate herd immunity threshold for *R*_o_ = 2.5 is 0.60, implying that 60% of the population is protected either by vaccination or previous exposure to establish herd immunity.

The government of Cape Verde has demonstrated political will and commitment to meeting the standards set by COVAX and ensuring the effective distribution of vaccines [16]. Public awareness campaigns have been instrumental in promoting vaccination, with a particular emphasis on proactive community outreach [5]. To facilitate the vaccination drive, the Digital Health Pass was developed in collaboration with the WHO to verify vaccination status [15]. Although possessing the pass is not mandatory in Cape Verde, it serves as a useful tool for international travel purposes.

## CHALLENGES TO HERD IMMUNITY

Cape Verde encountered several challenges in its efforts to distribute COVID‐19 vaccines, including difficulties in reaching remote areas, inadequate monitoring of vaccination data, and improper vaccine storage practices [23]. Vaccine supply remained a major obstacle to rapid distribution. Cape Verde received its first vaccine supply in March 2021, later than many other countries, highlighting supply security as a fundamental challenge [24]. In low‐income countries, such as Cape Verde, vaccine unavailability and high costs, coupled with financial gaps, hindered access to vaccines [9]. Although more doses arrived in May 2021, vaccination efforts initially focused on priority target groups [23].

Most available vaccines were only authorized for use in individuals aged 16 and above, necessitating continuous education and awareness campaigns among the adult population [25]. Vaccine availability and logistical issues posed a significant threat in low‐income countries like Cape Verde, with reports of vaccines nearing their expiry dates upon arrival due to the short shelf life of vaccines distributed through COVAX [9]. This resulted in vaccine wastage before reaching the intended areas of disbursement and collection, particularly in rural communities [25].

The emergence of new variants, such as the highly transmissible and vaccine‐resistant Omicron and its sub‐variants, further complicated the goal of achieving herd immunity [26]. The Delta variant was detected in the Santiago islands, which had the highest death rate and hospital visits in Cape Verde [27]. Additionally, the presence of the less virulent Alpha variant was also reported [27]. The need for booster shots and revaccination added logistical challenges to the vaccination efforts.

Workforce capacity was another obstacle, as deploying the existing health workforce for COVID‐19 vaccine delivery created a gap in the provision of other essential services crucial for population well‐being [15]. The health system's recovery from the pandemic resulted in an overburdened healthcare workforce. Prior disruptions to Cape Verde's economy caused by the pandemic, drought, and other global crises led to high levels of food insecurity, posing significant risks to the population, especially vulnerable groups [28]. With competing challenges, the COVID‐19 response gradually lost priority, resulting in reduced government and institutional focus on COVID‐19 management.

## RECOMMENDATIONS

To achieve herd immunity, stakeholders must collaborate and prioritize community‐based health systems. A comprehensive policy package is crucial to coordinate efforts in combating COVID‐19. Infrastructure, such as local health posts and community health workers, should be utilized to overcome vaccine delivery challenges, especially in remote areas. Establishing a community COVID‐19 vaccine task force, comprising leaders from various categories, is vital for effective advocacy and information dissemination. Low‐income countries should seek partnerships with international organizations, NGOs, and the private sector to enhance their vaccine delivery capacity.

Although transmission‐blocking vaccines are not currently available, the existing vaccines have demonstrated efficacy in reducing the severity of symptoms. However, the occurrence of vaccine supply disruptions exacerbates vaccine hesitancy and poses operational challenges. To reinforce healthcare systems, it is imperative to exhibit strong leadership, political engagement, allocate sufficient funding, and foster collaboration among stakeholders and sectors. By cooperating with neighboring countries, the cost of vaccine procurement and delivery can be minimized, whereas international partners can provide valuable technical assistance in enhancing disease surveillance and response systems.

The pharmaceutical industry plays a crucial role in enhancing health system resilience. Collaboration between the government and the pharmaceutical sector can establish more vaccination‐approved institutes, strengthen healthcare systems, educate healthcare professionals, and improve vaccine availability. Leveraging digital technology and innovation can enhance the productivity of the health sector and drive effective responses to achieve herd immunity. Developing a robust pharmacovigilance system that monitors vaccine safety is essential, including the collection and analysis of adverse effects nationwide. Strengthening STEM education will cultivate young talent to drive research and innovation in infectious disease response. Educational programs addressing COVID‐19 vaccine concerns, side effects, and misconceptions should be implemented to positively influence vaccine uptake and facilitate herd immunity.

## CONCLUSION

The COVID‐19 pandemic had a devastating impact on Cape Verde's health system and tourism sector. Nonetheless, the government of Cape Verde displayed exceptional leadership and commitment in effectively managing the crisis. They implemented a range of measures and strategies, including a successful vaccination program that resulted in high vaccination coverage. This achievement was made possible through the combined efforts of dedicated healthcare providers and active community participation. Although Cape Verde has achieved impressive results, there is still work to be done to maintain this success and tackle remaining challenges, such as ensuring equitable distribution of vaccines and addressing vaccine hesitancy.

## AUTHOR CONTRIBUTIONS

Conceptualization; data curation; project administration; writing—original draft; writing—review and editing: Deborah Oluwaseun Shomuyiwa. Conceptualization; data curation; writing—original draft: Morolake Ibukunoluwa Otitodun. Conceptualization; data curation; writing—original draft; writing—review and editing: Innocentia Tobechukwu Anyanwu. Writing—original draft; writing—review and editing: Muhsinah Adesewa Abdulwasiu. Writing—review and editing: Maria de Fátima Carvalho‐Alves and Maria da Luz Lima Mendoca. Conceptualization; project administration; writing—original draft; writing—review and editing: Goodness Ogeyi Odey. Writing—original draft: Alhaji Umar Sow.

## CONFLICT OF INTEREST STATEMENT

Deborah Oluwaseun Shomuyiwa, Goodness Ogeyi Odey are members of the Youth Editorial Board of Public Health Challenges and coauthors of this article. To minimize bias, they have been excluded from all editorial decision‐making related to the acceptance of this article for publication.

## FUNDING INFORMATION

The authors received no specific funding for this paper.

## Data Availability

No database or primary data were used in preparing the manuscript.
